# Obituary

**Published:** 1890-10

**Authors:** 


### Obituary.

Died, Sept. 8th, 1890, at the home of his brother in Georgetown, Brown County, Ohio, Dr. Wm. H. Woodward, son of Dr. C. N. Woodward, of Ripley, Ohio.

He had been afflicted for some months with cancer of the tongue, that terrible disease which in but a small proportion of cases yields to treatment. Dr. Woodward was a graduate of the Ohio College of Dental Surgery. Soon after his graduation he began the practice of his profession in Cincinnati, in which he was successfully engaged till within the last two months, his strength being so reduced he was compelled to relinquish business. He was a man of good ability, his manner was quiet and reserved, and on that account he was less widely known than many others of less ability. He was an active and honored member of the Knights Templar of which body he was a past eminent commander. He stood high in that body as he did in the esteem of all who had an intimate acquaintance with him.

Death of Dr. William Brodie.—Dr. William Brodie, of Detroit, died at his residence in that city on July 30. Few men in the medical profession were more widely known than Dr. Brodie. For more than a third of a century he had been an active member of the American Medical Association, and his loyalty to its interests was proverbial. Its highest honor was conferred when he was elected president. In the discharge of the duties in connection with that office his services commanded universal approval. It is difficult for the moment, as the news of his death comes to us over the wires, to realize that in the future meetings of the association his place will be evermore vacant. His relations with the association have been so inseparable that the record of his life becomes a part of its history, and as time permits other pens will do ample justice to his memory, —*Journal of the Amer. Med. Association.*

